# A Case of Ventral Spinal Cord Herniation from a Chronic Dural-pleural Fistula Resulting in Thoracic Myelopathy

**DOI:** 10.7759/cureus.6123

**Published:** 2019-11-11

**Authors:** Ilyas Eli, Jian Guan, Michael Karsy, Marcus D Mazur, Andrew Dailey

**Affiliations:** 1 Neurological Surgery, University of Utah School of Medicine, Salt Lake City, USA

**Keywords:** dural-pleural fistula, cerebrospinal fluid leak, myelopathy

## Abstract

Formation of a dural-pleural fistula is uncommon after anterior thoracic spine surgery, tumor, or trauma. The goal of surgical management is to terminate the connection between the pleura and subarachnoid space. We describe a case of chronic dural-pleural fistula in a 70-year-old woman and present a unique surgical treatment option. The patient presented 25 years after an anterior thoracic surgery she had undergone for a thoracic disc herniation, with a dural-pleural fistula and ventral herniation of the spinal cord into the defect. She was treated with a bovine pericardium sling patch to cover the defect. This case highlights the identification of a chronic thoracic dural-pleural fistula and surgical treatment with double intradural and extradural layering of bovine pericardium sling patch, which has not been described previously for chronic thoracic dural-pleural fistula.

## Introduction

Thoracic dural-pleural fistula is rare a condition caused by injuries to the dura, which results in cerebrospinal fluid (CSF) leaking into the pleural cavity and leading to fistula formation. Development of the fistula is related to abnormal communication between the pleura and thoracic dura due to complications related to trauma, malignancy, or anterior or anterolateral approaches to the thoracic spine [1]. Negative pressure within the pleura induces a pressure differential that allows flow from the higher pressure subarachnoid space into a low-pressure pleura [2]. Dural-pleural fistulas can result in the formation of pleural effusion and intracranial hypotension as a result of CSF drainage into the pleural cavity. Without treatment, the fistulous connection persists, requiring surgical repair.

To the best of our knowledge, a chronic thoracic dural-pleural fistula has only been reported once previously. In that case, the fistula was repaired with an omental flap. Here we report the case of a patient with chronic thoracic dural-pleural fistula who presented with thoracic myelopathy secondary to spinal cord herniation into the fistulous defect. We describe our surgical technique in which the fistula was treated with a bovine pericardium sling patch.

## Case presentation

A 70-year-old woman presented to the clinic with the chief complaints of progressively worsening back pain and weakness in her legs. She had progressively worsening symptoms of thoracic myelopathy of 2 years' duration. She described the lower back pain as radiating to both of her legs. She had a walking tolerance of <10 feet and reported severe sensory disturbances with loss of lower-extremity proprioception that had resulted in multiple falls. She had eventually become wheelchair-bound because of the lower extremity weakness. Interestingly, over the previous three years, she had been hospitalized three times for left-sided pleural effusions and pneumonia. Additionally, she had had an intrathecal pain pump placed to alleviate the pain.

The patient had a long history of spine-related problems. At the age of 45 years, she had undergone an operation for removal of a herniated thoracic disk at T8-9 after which she had developed a pleural-CSF fistula, which had been treated with a patch placement. Further details regarding the operation were unclear, but the surgery had been performed via thoracotomy for resection of the thoracic disk herniation. Five years later, another pleural-CSF fistula had developed, and a patch had been surgically placed a second time at T8-9. She had also previously undergone anterior cervical discectomy and fusion at C3-7 and posterior spinal fusion at C3-T1.

A physical examination demonstrated that she had diffusely decreased strength in her bilateral lower extremities with 4/5 strength in hip and knee flexion and extension and 3/5 strength in plantar flexion and dorsiflexion. The sensation was decreased throughout the lower extremities in a non-radicular distribution. No clonus was noted and the tone was normal. Her other neurological examination results were normal.

**Figure 1:**
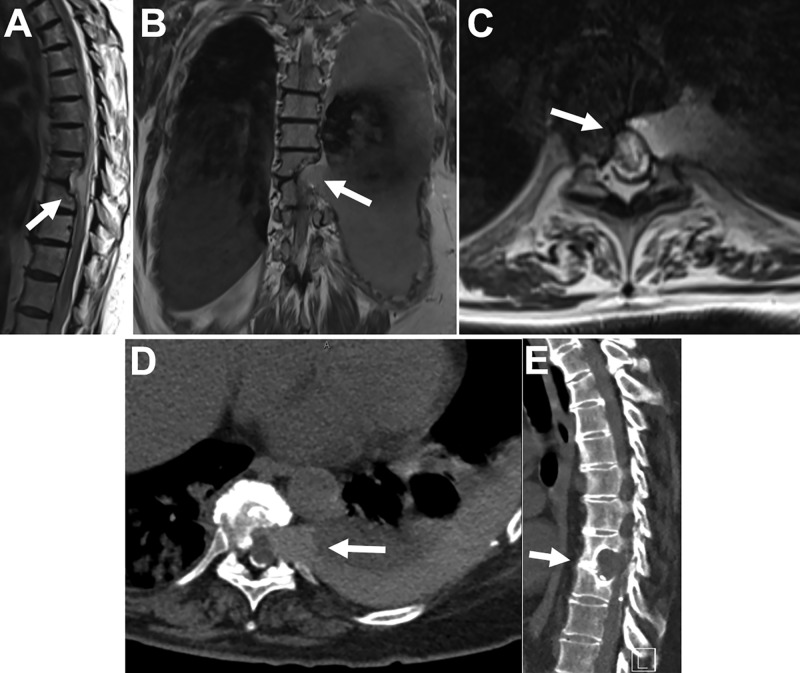
Preoperative MRI and CT imaging of the thoracic spine (A) sagittal T2-weighted MRI demonstrating a ventrolateral defect at the left T8-9 level (arrow). (B) coronal T2-weighted MRI showing herniation of the pleural cavity into the surgical defect (arrow) and the presence of a pleural effusion. (C) axial T2-weighted MRI showing displacement of the ventral spinal cord into the defect and contact of the herniated pleura with the thecal sac contents (arrow). (D) axial CT myelogram demonstrating a hyperdense fluid extending from the spinal canal to adjacent pleural space consistent with a dural-pleural fistula (arrow). (E) sagittal CT image of the thoracic spine demonstrating the osseous defect (arrow)

An MRI of the thoracic spine without contrast enhancement demonstrated a partially resected vertebral body on the left T8-9 level (Figure 1). The spinal cord was displaced ventrally, with herniation of the thecal sac into the defect and in contact with the area of pleural herniation. There was evidence of myelomalacia of the spinal cord from T5 to T10, which was most severe in the area of herniation at T8-T9. A CT myelogram demonstrated contiguity of the left pleural space with the spinal canal at T8-9 (Figure 1).

**Figure 2:**
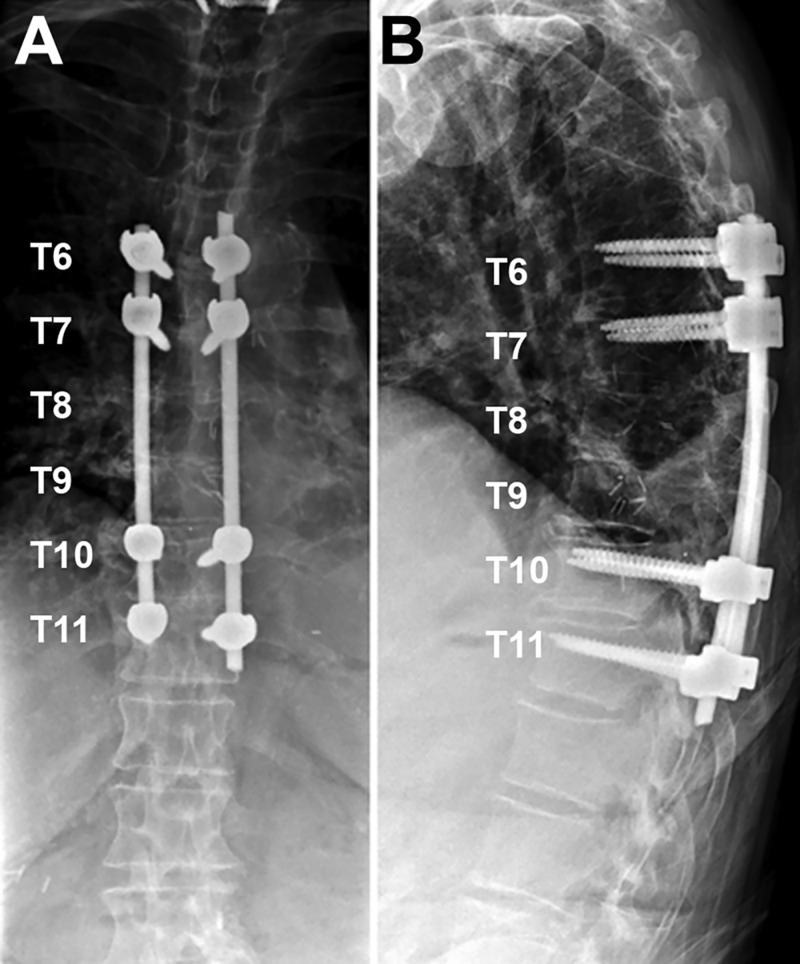
Postoperative X-rays demonstrating T6-11 posterior spinal fusion (A) anteroposterior X-ray. (B) lateral thoracic X-ray

**Figure 3:**
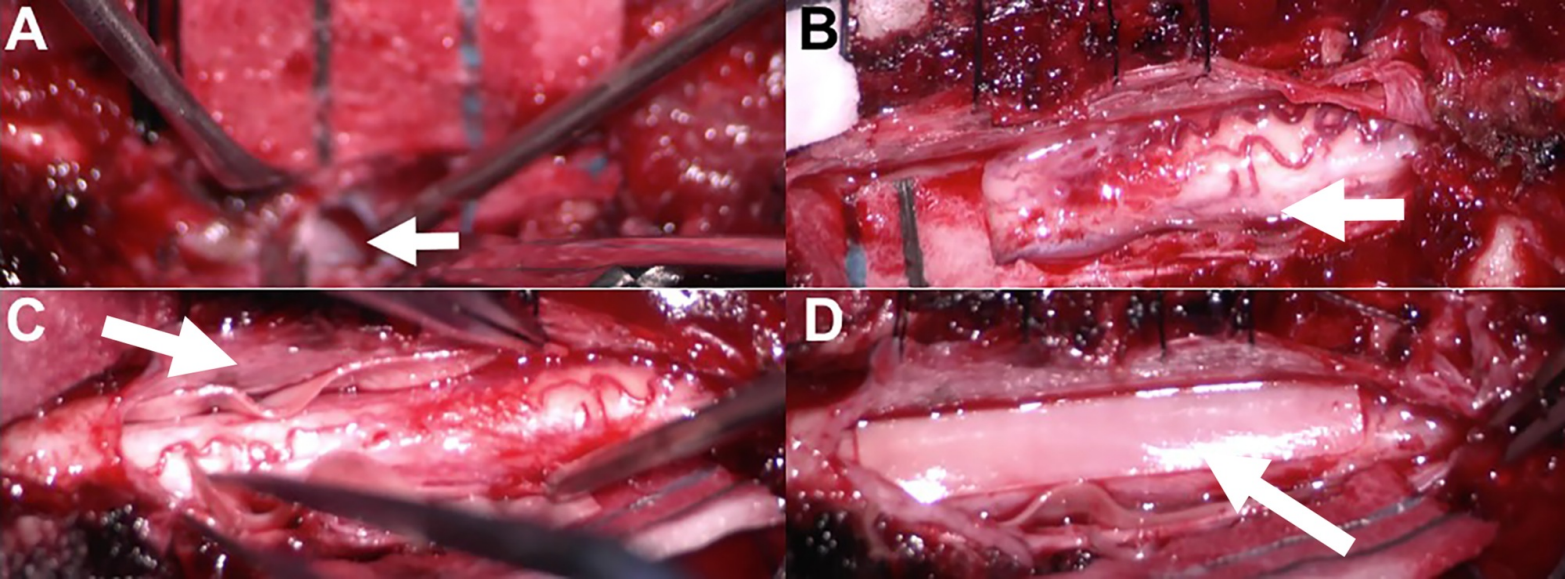
Intraoperative images (A) the ventrolateral defect signified by the arrow. (B) the exposure of the spinal cord. (C, D) the placement of a bovine pericardium sling patch to circumferentially cover the fistula

The patient underwent laminectomy from T7 to T9 along with the drilling of the left T8 and T9 pedicle and transverse process to expose the lateral margin of the dura. This was followed by a T6-T11 posterior segmental instrumentation, excluding pedicle screw placement at T8 and T9 (Figure 2). During the exploration of the lateral margin of the dura, a large defect was found where the spinal cord was herniating into the ventral defect, with clear communication between the subarachnoid space and thorax. The dura was opened and there was clear evidence of communication of the subarachnoid space to the fistulous space with drainage of spinal fluid into the thorax. We performed intradural lysis of adhesions. The spinal cord was dissected free anteriorly from the dura and mobilized. Once the spinal cord was completely dissected free of the anterior margin of the dural defect, a bovine pericardial sling was measured and cut to cover the defect. The sling was then placed into a position anterior to the spinal cord. A lumbar drain was placed under direct visualization and then the dura was closed. An additional piece of the bovine pericardium was placed to bolster the anterior portion of the defect. Additionally, a small bovine pericardium patch was placed over the fistulous connection. The patches were secured with Tisseel fibrin sealant (Baxter Healthcare, Deerfield, IL) anteriorly (Figure 3). The anterior defect was further secured with muscle and the dorsal dural defect was covered with Surgicel (Ethicon Inc, Somerville, NJ) and Tisseel. No lumbar drain was placed.

The patient had a protracted stay in the intensive care unit with the left pneumothorax requiring a chest tube and non-CSF pleural effusion. The chest tube and lumbar drain were both removed on postoperative day seven, and the patient was discharged to inpatient rehabilitation on postoperative day 15. The patient has reported that she continues to get physical therapy 1 year postoperatively. She continues to gain strength and is now ambulatory.

## Discussion

One rare complication of anterior approaches to the thoracic spine is the formation of a dural-pleural fistula [3]. A fistula forms when a connection develops between the pleural cavity and the subarachnoid space, which allows the flow of CSF into the pleura. Patients can present with pleural effusion symptoms of respiratory distress, dyspnea, or hypoxia. Other presentations include intracranial hypotension with symptoms of postural headache, nausea, vomiting, and vertigo [3]. The pathophysiology involves a tear in the dura and arachnoid and opening of the parietal pleura [4]. The negative intrapleural pressure acts to suction CSF flow, which is augmented by CSF pulsations, resulting in a flow through the fistula, which prevents the closure of the dura [5,6]. The herniated spinal cord in our case underwent continual strangulation as it coursed through a limited space, resulting in cord compression and myelopathy.

Surgeons should be wary of this complication in their patients who undergo thoracic spine surgery. Early diagnosis can prevent the development of progressive myelopathy and meningitis. In patients with pertinent symptoms, the diagnosis can be achieved by sampling the pleural effusion if present and testing for b2-transferrin, a test with 100% sensitivity and 95% specificity [7,8]. Imaging with MRI or CT myelography is a simpler diagnostic tool. Myelographic studies provide anatomic visualization of the specific location of the fistula. A T2-weighted MRI is a less invasive, but less sensitive alternative [9].

Spontaneous resolution of the dural-pleural fistula is very unlikely, and surgical intervention is the definitive treatment for it. A wide range of treatment options has been published in the literature, with most describing repair of the pleural and dural defects. Interventions include primary repair or use of cadaveric graft, muscle flaps, or omental flaps [2,4,6,10,11]. The repair can be augmented with a secondary layer of sealants such as fibrin glue, epidural blood patch, Tisseel, DuraSeal (Integra Lifesciences, Plainsboro, NJ), or Onyx (Medtronic-Covidien, Minneapolis, MN) [1,6,8,12]. Alternatively, noninvasive positive pressure mask ventilation combined with chest tube drainage can be used to increase intrathoracic pressure, which decreases CSF flow by decreasing the pressure gradient between the subarachnoid and pleural spaces [5,13].

Most cases of dural-pleural fistulas reported in the literature have been treated soon after the complication was noted. Our patient’s course may represent the natural history and progression of this complication, with spinal cord herniation resulting when the fistula is not repaired early. Because our patient’s repeated bouts of pneumonia represented a risk for meningitis, the combined risk of meningitis and myelopathy indicated the need for aggressive treatment. Sahota et al. [2] have described a dural-pleural fistula repair with a tunneled vascularized flap via a laparotomy. The use of an omental flap requires a laparotomy, which can lead to intra-abdominal complications, prolonged surgery, and respiratory complication. Although the closure of the anterior dural defect with duraplasty has been described for the closure of idiopathic spinal cord herniation, this technique has not been implemented for the treatment of dural-pleural fistulas [14-16]. We used an intradural approach commencing with lysis of intradural adhesions and dissecting the spinal cord free from the dura. Then, a bovine pericardial patch was placed circumferentially underneath the spinal cord in a sling fashion. The sling allows for complete coverage of the defect by maintaining the spinal cord intradurally to prevent re-herniation. The sling is not watertight but will heal and close the dural defect over time. After the closure of the dura, to further bolster the closure, another bovine pericardial patch and piece of muscle were placed over the hernial defect extradurally. Additionally, the insertion of a subarachnoid thoracic drain allowed for CSF diversion while the patch healed over the adjacent dura for complete obliteration of the fistula. The advantages of this double-layering technique include direct visualization and coverage of the defect with a multi-layer bovine patch, which promotes healing of the region and allows closure of the dural-pleural fistula.

## Conclusions

In this case, we treated a chronic dural-pleural fistula in the thoracic spine associated with spinal cord herniation with a meticulous closure of the defect via a double-layering of intradural and extradural bovine pericardium sling patch, watertight dural closure, muscle, surgical glue, and a subarachnoid thoracic drain for CSF diversion. Our technique provides an efficient closure of the defect and avoids the complications associated with omental flaps. Although chronic dural-pleural fistulas are rare, a comprehensive understanding of its pathophysiology and surgical treatment options will assist spine surgeons in its diagnosis and treatment if encountered.
